# Soft Tissue Metastases as the First Clinical Manifestation of Squamous Cell Carcinoma of the Esophagus: Case Report

**DOI:** 10.4021/wjon2010.05.209w

**Published:** 2010-05-19

**Authors:** Manish Chand, Rachael J. Thomas, Natalie Dabbas, Adrian C. Bateman, Gavin T. Royle

**Affiliations:** a Basingstoke and North Hampshire NHS Foundation Trust, Aldermaston Road, Basingstoke, Hampshire RG24 9NA, UK; b Queen Alexandra Hospital, Cosham, Portsmouth PO6 3LY, UK; c Salisbury District Hospital, Salisbury, Wiltshire SP2 8BJ, UK; d Southampton University Hospitals NHS Trust, Tremona Road, Southampton, Hampshire SO16 6YD, UK

**Keywords:** Soft tissue metastasis, Esophagus, Squamous cell carcinoma, Occult cancer, Biopsy

## Abstract

Soft tissue metastases are an uncommon presenting feature for primary solid tumours. This case highlights a rare presentation in which a soft tissue mass is the first clinical manifestation of a widespread disseminated malignancy of the esophagus. A 73-year-old woman presented with a soft swelling in the left upper quadrant of the abdomen arising from the anterior abdominal wall, suspicious of liposarcoma. Core biopsies revealed squamous carcinoma. Immunohistochemistry suggested the most likely diagnosis was that of metastatic carcinoma with a number of potential primary sites. Computed tomography scanning showed widespread metastatic disease, including lung, liver, kidney, omentum, subcutaneous and intramuscular lesions. The distal esophagus was noted to be circumferentially thickened. Finally, upper gastrointestinal endoscopy revealed carcinoma of the esophagus. The patient remains well awaiting esophageal stenting and palliative chemotherapy. In conclusion, it is important to be able to distinguish the origin of a soft-tissue swelling as the management will depend significantly on the histological type. Soft-tissue metastases are rarely encountered as a presenting sign of an occult cancer. Primary cancers that most commonly metastasise to soft tissues include those arising within the lung, colon and kidney. The most frequent histological diagnosis is adenocarcinoma. This case demonstrates the utility of biopsy in the investigation of soft tissue masses when the clinical presentation is unusual.

## Introduction

Soft tissue metastases are an uncommon presenting feature for primary solid tumours [1-3] and represent less than 3% of soft-tissue malignancies [4]. It is important to distinguish such metastases from a soft-tissue sarcoma as they may represent the first clinical sign of an occult tumour. A combination of thorough clinical assessment, appropriate imaging studies and tissue biopsies with appropriate immunohistochemical techniques are required to accurately reach a diagnosis.

We present a unique case of an elderly woman who presented with a short history of a left-sided abdominal wall swelling. Further investigation with a series of imaging studies, tissue biopsies and endoscopy revealed widespread visceral and soft tissue metastases arising from a squamous cell carcinoma of the esophagus. This case highlights a rare presentation in which a soft tissue mass is the first clinical manifestation of a widespread disseminated malignancy of the esophagus: an aggressive cancer which has a poor prognosis owing to its late presentation.

## Case Report

A 73-year-old woman was referred to a ‘Sarcoma’ clinic suspected of having a soft-tissue malignancy. The woman had noticed a slow-growing lump on the left side of her abdomen, which had been present for 2 months. The abdominal lump was her primary complaint and she had no significant past medical history. On examination she was noted to have a soft swelling in the left upper quadrant of the abdomen, which felt as if it was arising from the anterior abdominal wall.

**Figure 1:**
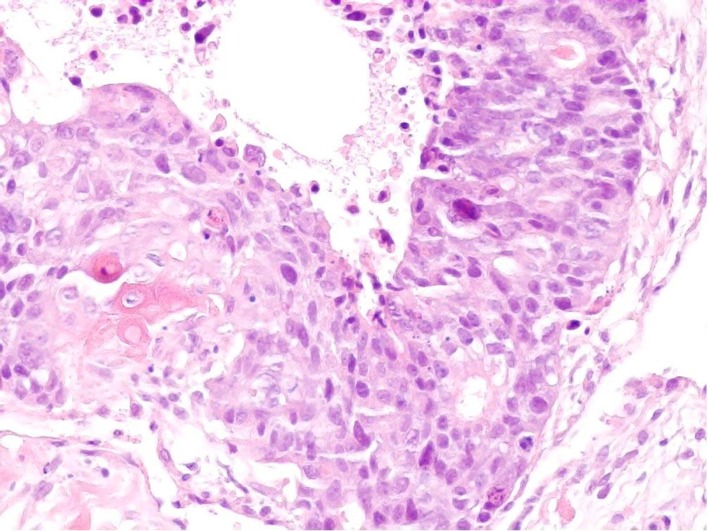
Core biopsy of anterior abdominal wall lesion. H and E stain (x 200).

An ultrasound scan of the abdomen wall demonstrated a complex mass lying within the fatty layer. The lesion extended to 3.5 cm in diameter with a central lobular, heterogenous portion surrounded by fat, which demonstrated marked internal vascularity on Doppler study. This was suspicious of liposarcoma, and 3 core biopsies of the lump were taken which revealed extensively necrotic moderately to poorly differentiated carcinoma. There were both glandular and focal squamous differentiation histologically with keratinisation. Figure 1 illustrates histology from the core biopsy obtained from this abdominal wall lesion. The photomicrograph shows part of a malignant epithelial tumour exhibiting both squamous (left side) and glandular (right side) differentiation. Immunohistochemistry showed focal expression of cytokeratin 20 and a rare cell showing cytokeratin 7. TTF1 (a marker of primary thyroid and non-small cell lung carcinoma), oestrogen receptor and neuroendocrine markers was not expressed. At this stage, the differential diagnosis included a primary skin adnexal carcinoma, for example, a sweat gland carcinoma; however, the histology did not show the classical pattern of the best-recognised subtypes. Therefore, the more likely diagnosis was that of metastatic carcinoma with a number of potential primary sites. A computed tomography (CT) scan and upper gastrointestinal (GI) endoscopy were therefore arranged.

**Figure 2:**
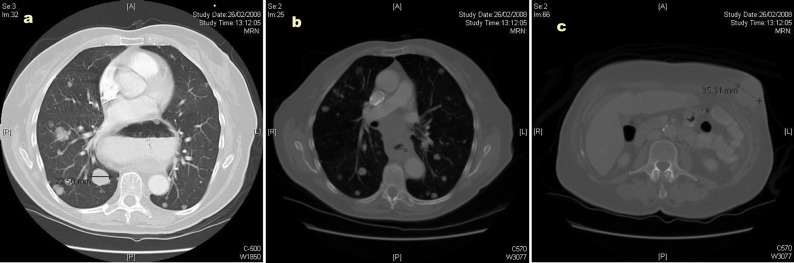
(a) Computed tomography (CT) image illustrating multiple lung metastases; (b) Computed tomography image illustrating circumferential esophageal wall thickening; (c) Computed tomography image illustrating the anterior abdominal wall lesion.

The CT scan showed widespread metastatic disease. Beginning cranially, within the lungs there were multiple rounded nodules of varying size, the largest measuring 23 mm, consistent with metastases. In the right superior mediastinum there was an enlarged node measuring over 2 cm. This node was separated from the right lobe of the thyroid by the jugular vein but posteriorly shown to be inseparable from the upper thoracic esophagus. The distal esophagus and gastro-esophageal junction was noted to be abnormally circumferentially thickened and irregular; however, there was no proximal dilatation of the esophagus. There were further metastatic deposits in the liver, kidneys and omentum. A number of subcutaneous and intramuscular lesions were identified throughout the thorax and abdomen, but no lytic bone lesions. The original left abdominal mass was identified as originating from the anterior abdominal wall and extending into the subcutaneous tissue: Figures 2a, b and c illustrate CT images of multiple lung metastases (the largest measuring 22.50 mm), of the circumferential esophageal wall thickening consistent with the primary tumour, and of the anterior abdominal wall lesion, respectively.

An upper GI endoscopy revealed an extensive, friable and bleeding carcinoma of the esophagus at between 25 cm and 31 cm. Biopsies of the lesion revealed squamous cell carcinoma. The biopsies of the abdominal wall and the esophageal lesion were compared and confirmed as showing identical pattern.

The patient was subsequently referred to the upper GI cancer team and at the time of writing, despite the substantial tumour burden, remains well awaiting esophageal stenting and palliative chemotherapy.

## Discussion

It is important to be able to distinguish the origin of a soft-tissue swelling as the management will depend significantly on the histological type. Although not in themselves infrequent, soft-tissue metastases are rarely encountered as a presenting sign of an occult cancer as by the time these appear, the patient has commonly developed symptoms pertaining to the primary tumour. Primary cancers that most commonly metastasise to soft tissues include those arising within the lung, colon and kidney [5-7]. Further, the most frequent histological diagnosis is adenocarcinoma [8].

The prognosis and treatment of esophageal cancer is dictated by the extent of local invasion, but more importantly by both nodal and metastatic spread. Early detection with surgical resection and appropriate adjuvant therapy confers the best chance of survival; however, by the time most patients who present this may not be feasible. Palliative techniques such as esophageal stenting with self-expanding metallic stents (SEMS) may provide symptomatic relief from dysphagia. Initial staging of esophageal cancer involves CT scanning and endoscopic ultrasound, although PET scans have been suggested to be more useful in detecting soft-tissue metastases [9].

There are few reported cases of metastatic spread of esophageal cancer to soft-tissues and all were known cases of primary cancer. As far as the authors are aware, there are no reported cases of soft-tissue metastases presenting as the first clinical sign of an underlying squamous cell carcinoma of the esophagus.

In conclusion, it is all too easy to attribute superficial soft tissue swellings to common conditions and plan excision based on the likeliest diagnosis. As shown in the case described, there is no substitute for thorough clinical questioning and examination, and any disparity between the clinical presentation and examination findings should prompt additional investigation before further management can be appropriately planned. This case also demonstrates the utility of biopsy in the investigation of soft tissue masses when the clinical presentation is unusual.

## References

[1] Leinung S, Mobius C, Udelnow A, Hauss J, Wurl P (2007). Histopathological outcome of 597 isolated soft tissue tumors suspected of soft tissue sarcoma: a single-center 12-year experience. Eur J Surg Oncol.

[2] Glockner JF, White LM, Sundaram M, McDonald DJ (2000). Unsuspected metastases presenting as solitary soft tissue lesions: a fourteen-year review. Skeletal Radiol.

[3] Damron TA, Heiner J (2000). Distant soft tissue metastases: a series of 30 new patients and 91 cases from the literature. Ann Surg Oncol.

[4] Sudo A, Ogihara Y, Shiokawa Y, Fujinami S, Sekiguchi S Intramuscular metastasis of carcinoma. Clin Orthop Relat Res.

[5] Bibi C, Benmeir P, Maor E, Sagi A (1993). Hand metastasis from renal cell carcinoma with no bone involvement. Ann Plast Surg.

[6] Laurence AE, Murray AJ (1970). Metastasis in skeletal muscle secondary to carcinoma of the colon—presentation of two cases. Br J Surg.

[7] McKeown PP, Conant P, Auerbach LE (1996). Squamous cell carcinoma of the lung: an unusual metastasis to pectoralis muscle. Ann Thorac Surg.

[8] Plaza JA, Perez-Montiel D, Mayerson J, Morrison C, Suster S (2008). Metastases to soft tissue: a review of 118 cases over a 30-year period. Cancer.

[9] Kozyreva ON, Mezentsev DA, King DR, Gomez-Fernandez CR, Ardalan B, Livingstone AS (2007). Asymptomatic muscle metastases from esophageal adenocarcinoma. J Clin Oncol.

